# 晚期非小细胞肺癌表皮生长因子受体基因突变情况和其对吉非替尼疗效的影响

**DOI:** 10.3779/j.issn.1009-3419.2012.09.03

**Published:** 2012-09-20

**Authors:** 巍 钟, 孟昭 王, 龙芸 李, 莹 夏, 闽江 陈, 力 张, 静 赵

**Affiliations:** 100730 北京，中国医学科学院北京协和医学院北京协和医院呼吸内科 Department of Respiratory Diseases, Peking Union Medical College Hospital, Peking Union Medical College and Chinese Academy of Medical Sciences, Beijing 100730, China

**Keywords:** 肺肿瘤, 表皮生长因子受体, 突变, 吉非替尼, Lung neoplasms, Epidermal growth factor receptor, Mutation, Gefitinib

## Abstract

**背景与目的:**

研究晚期非小细胞肺癌（non-small cell lung cancer, NSCLC）表皮生长因子受体（epidermal growth factor receptor, EGFR）基因突变情况和该基因突变状态对吉非替尼疗效的影响。

**方法:**

于2007年1月-2009年12月对160例晚期非鳞癌NSCLC患者进行了*EGFR*基因检测，*EGFR*基因外显子19和外显子21突变检测采用突变富集PCR法。其中111例接受了吉非替尼治疗。中位生存期（overall survival, OS）和无疾病进展生存时间（progression free survival, PFS）的比较采用*Kaplan-Meier*方法计算。

**结果:**

晚期非鳞癌NSCLC患者*EGFR*基因突变率为55%，多因素分析显示只有病理类型与是否突变明显相关。*EGFR*基因突变型患者的OS为29.0个月（95%CI: 24.2-33.8），野生型为21.0个月（95%CI: 14.7-27.3），两者差别无统计学差异。*EGFR*基因突变患者的PFS为17.0个月（95%CI: 5.6-17.6），而野生型为11.6个月（95%CI: 8.6-25.4），两者有明显性差别（*P*=0.022）。OS的多因素分析结果显示，OS与ECOG评分、病理类型、*EGFR*基因突变状态明显相关。PFS多因素分析结果显示，PFS与ECOG评分、既往化疗方案数和*EGFR*基因突变明显相关。*EGFR*基因外显子19突变与外显子21突变的OS和PFS无明显差别，客观疗效也无差别。

**结论:**

晚期非鳞癌NSCLC *EGFR*基因突变患者的PFS明显优于野生型患者，OS有延长趋势。*EGFR*基因不同突变类型的PFS和OS均无差别。

## Abstract (en)

**Background and objective:**

It has been proven that the status of epidermal growth factor receptor (EGFR) gene mutation was related to effects of gefitinib in patients with advanced non-small cell lung cancer (NSCLC). The aim of this study is to reports distribution of *EGFR* gene mutations in advanced NSCLC and their influence on effect of gefitinib.

**Methods:**

From Jan 2007 to Dec 2009, 160 patients with advanced non-squamous NSCLC received *EGFR* mutation tests, and *EGFR* exon 19 and 21 were amplified by mutant-enriched PCR and analyzed by sequencing. Among those patients, 111 received gefitinib therapy. Overall survival (OS) and progression free survival (PFS) were calculated using the *Kaplan-Meier* method and a *Cox* regression analysis was used to detect differences between strata.

**Results:**

The percentage of *EGFR* mutation in advanced non-squamous NSCLC was 55%, and it was only significantly related with pathological type. OS of the patients with or without *EGFR* gene mutations were 29.0 months (95%CI: 24.2-33.8) and 21.0 months (95%CI: 14.7-27.3) respectively, and the difference was not significant. PFS of patients with or without *EGFR* gene mutations were 17.0 months (95%CI: 5.6-17.6) and 11.6 months (95% CI 8.6~25.4), and the difference was significant (*P*=0.022). Multivariate analysis shows that OS was significantly related with ECOG status, pathological type and *EGFR* mutation statuses, and PFS was significantly related to ECOG status, former regimens number and *EGFR* mutation statuses. There were no significant differences in OS and PFS between patients with *EGFR* exon 19 deletions and those with exon 21 point mutation.

**Conclusion:**

PFS of patients with *EGFR* mutations was better than those without *EGFR* mutations, but OS was similar. There were no significant differences in OS and PFS between patients with *EGFR* exon 19 deletions and those with exon 21 point mutation.

晚期非小细胞肺癌（non-small cell lung cancer, NSCLC）患者的治疗主要以化疗为主，包括一线化疗和二线化疗，可以延长患者的生存期和改善生活质量。近年来，表皮生长因子受体酪氨酸激酶抑制剂（epidermal growth factor receptor tyrosine kinase inhibitor, EGFR-TKI）的出现使患者生存期进一步延长，甚至部分患者可以长期生存。研究^[1, 2]^表明EGFR-TKI的疗效与*EGFR*基因的突变情况明显相关，特别是外显子19的缺失突变和外显子21的点突变。这两种突变占所有敏感突变的90%以上。本研究回顾性地总结了北京协和医院2007年1月-2009年12月3年间收治的NSCLC患者*EGFR*基因检测结果和基因突变对吉非替尼治疗疗效和不良反应的影响。

## 1 对象和方法

### 1.1 对象

均为就诊于北京协医院的患者，符合以下条件：≥18岁；均有组织学证实为非鳞癌的NSCLC；有足够的组织标本进行*EGFR*基因突变检测；临床分期为Ⅲb期或Ⅳ期；患者病例资料完整。共收集患者160例，其中111例接受了吉非替尼治疗。

### 1.2 试验方法

收集患者的临床资料，包括所有患者的性别、年龄、吸烟情况、组织类型和ECOG体能评分。对于接受吉非替尼治疗的患者收集病例相关资料，包括：既往化疗方案数，是否有骨转移、脑转移、肝脏转移、其它肺叶转移和胸腔积液，吉非替尼治疗的客观疗效、疾病进展时间和死亡时间以及治疗相关的不良反应（包括皮疹、腹泻、食欲下降、肝脏转氨酶升高和恶心/呕吐等）。客观疗效评价根据实体瘤疗效评价标准（Response Evalutation Criteria in Solid Tumor, RECIST）进行评估。无疾病进展生存期（progression free survival, PFS）定义为从开始服用吉非替尼到疾病进展或患者死亡的时间（月），生存期（overall survival, OS）定义为从开始服用吉非替尼到患者死亡的时间（月）。

### 1.3 *EGFR*基因检测方法

所有组织标本均为患者进行治疗前经病理科医生确定为NSCLC的组织石蜡包埋切片。本研究采用限制性内切酶法联合巢式PCR法对*EGFR*基因外显子19和21进行突变检测，该法之前已经进行了报道^[3]^。本研究采用的方法对之前方法稍作改进。肿瘤DNA提取采用磁珠法（北京金麦格生物技术有限公司），引物序列（表 1）由上海Invitrogen公司合成。最终PCR产物由上海Invitrogen公司完成测序，使用DNAMAN软件阅读序列。

**1:** 突变富集PCR引物、复性温度及产物长度 The ME-PCR primers, annealing temperatures and the lengths of the products

Exon	Primer	Primer sequences	Tm (℃)	Fragment
19	Outer	Forward: 5’-ATCCCAGAAGGTGAGAAAGATAAAATTC -3’	63.2	204 bp[WT]
		Inverse: 5’-ACATTTAGGATGTGGAGATGAGCAG-3’	62.8	
	Inner	Forward: 5’-AGGTGAGAAAGATAAAATTCCCGTC-3’	62.0	182 bp[WT]
		Inverse: 5’-GAGATGAGCAGGGTCTAGAGCAG-3’	61.9	
21	Outer	Forward: 5’-TCAGAGCCTGGCATGAACATGACCCTG-3’	74.6	297 bp
		Inverse: 5’-GGTCCCTGGTGTCAGGAAAATGCTGG-3’	73.0	
	Inner	Forward: 5’-CAGCAGGGTCTTCTCTGTTTC-3’	59.1	213 bp
		Inverse: 5’-GAAAATGCTGGCTGACCTAAAG-3’	60.3	

巢式PCR及酶切条件如下：①巢式PCR第一轮（PCR1）：反应体系：10 μL，包括DNA聚合酶及反应混合物GoTaq mix 5 μL、上下游引物（10 pm/μL）各0.5 μL、基因组DNA 5 ng-100 ng，无核酸水补齐至10 μL；反应条件：95 ℃ 5 min；95 ℃ 30 s，60 ℃ 30 s，72 ℃ 30 s，20个循环；72 ℃ 10 min。②酶切富集突变（参见MseI或MscI说明书）：体系为20 μL，取1 μL第一轮PCR反应产物，使用MseI（对于外显子19）或MscI（对于外显子21）0.5 μL，10×Buffer 1 μL，无核酸水补齐至20 μL；酶切条件：37 ℃ 4 h。③巢式PCR第二轮（PCR2）：反应体系：50 μL，包括DNA聚合酶及反应混合物GoTaq mix 25 μL、上下游引物（10 pm/μL）各3 μL、模板DNA（即PCR1产物酶切后的DNA）2 μL，核酸游离水补齐至50μL；反应条件：95 ℃ 5 min；95 ℃ 30 s，60 ℃ 30 s，72 ℃ 30 s，40个循环；72 ℃ 10 min。

### 1.4 统计方法

使用SPSS 16.0软件进行统计分析。各组患者中位OS和中位PFS的比较采用*Kaplan-Meier*方法计算。采用多因素*Cox*回归分析各种因素对生存期的影响，包括性别、年龄、吸烟状况、ECOG评分、病理类型、既往化疗方案数、骨转移、脑转移、肝脏转移、其它肺叶转移、胸腔积液和*EGFR*基因突变情况。计数资料采用*χ*^2^检验分析包括不同亚组患者*EGFR*基因突变率的差异和不同亚组吉非替尼治疗的客观疗效，多因素分析采用线性回归的方法进行分析，*P* < 0.05为差异有统计学意义。

## 2 结果

### 2.1 一般情况

2007年1月-2009年12月共160例非鳞癌的NSCLC患者接受了*EGFR*基因突变的检测，其中男性81例，女性79例；年龄 < 60岁91例，≥60岁69例；吸烟58例，不吸烟102例；ECOG 0分-1分119例，≥2分41例；非腺癌17例，腺癌143例。111例接受了吉非替尼治疗，其中15例为一线治疗，96例为二线治疗。

### 2.2 *EGFR*基因突变与临床特征的关系

160例患者共检测到*EGFR*基因突变88例（55%），其与患者临床特征的关系见表 2，可见是否有*EGFR*基因突变只与病理类型相关。多因素分析也显示只有病理类型与是否有突变明显相关（*χ*^2^=6.374, *P*=0.023），而其它因素无统计学意义。88例*EGFR*基因突变中，外显子19缺失突变41例，外显子21点突变47例。突变类型与患者临床特征的关系见表 3，单因素分析和多因素分析均未见任何患者临床特征与突变类型有关。

**2:** *EGFR*基因突变与患者临床特征的关系 The relationship of *EGFR* mutation statuses and clinical characteristics

Characteristics		*EGFR* mutation status		*χ*^2^	*P*
		Negative	Positive		
Gender				2.091	0.099
	Male	41	40		
	Female	31	48		
Age				2.750	0.207
	≤60	44	47		
	> 60	28	41		
Smoking history				2.624	0.073
	Ever	31	27		
	Never	41	61		
Pathology				6.374	0.023
	Adenocarcinoma	60	83		
	Non-adenocarcinoma	12	5		
ECOG PS				0.040	0.492
	0-1	53	66		
	2-4	19	22		
PS: performance status; EGFR: epidermal growth factor receptor.					

**3:** 各种*EGFR*基因突变类型的临床特征 The clinical characteristics of patients with *EGFR* exon 19 and 21 mutations

Characteristics		*EGFR* mutation type		*χ*^2^	*P*
		Exon 19	Exon 21		
Gender				1.029	0.212
	Male	21	19		
	Female	20	28		
Age				1.766	0.132
	≤60	25	22		
	> 60	16	25		
Smoking history				0.072	0.486
	Ever	12	15		
	Never	29	32		
Pathology				2.378	0.141
	Adenocarcinoma	37	46		
	Non-adenocarcinoma	4	1		
ECOG PS				1.233	0.194
	0-1	33	33		
	2-4	8	14		

### 2.3 *EGFR*基因突变与OS和PFS的关系

*EGFR*基因突变患者的OS为29.0个月（95%CI: 24.2-33.8）；而野生型患者的OS为21.0个月（95%CI: 14.7-27.3），两者差异无统计学意义（*P*=0.066）（图 1）。*EGFR*基因突变患者的PFS为17.0个月（95%CI: 5.6-17.6）；而野生型患者的PFS为11.6个月（95%CI: 8.6-25.4），两者有明显差异（*P*=0.022）（图 2）。

**1:**
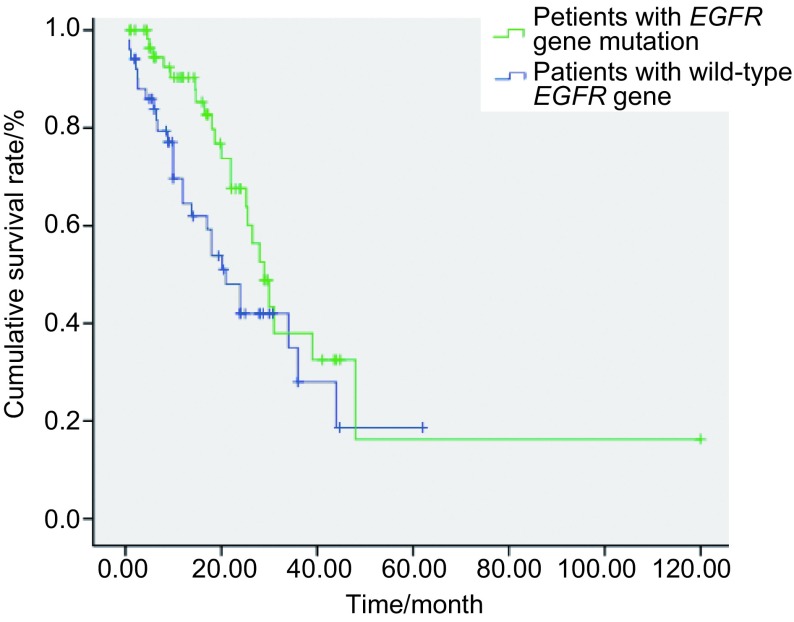
*EGFR*基因野生型和突变型患者的OS曲线 The OS curves of the *EGFR* mutation positive and negative patients. OS: overall survival.

**2:**
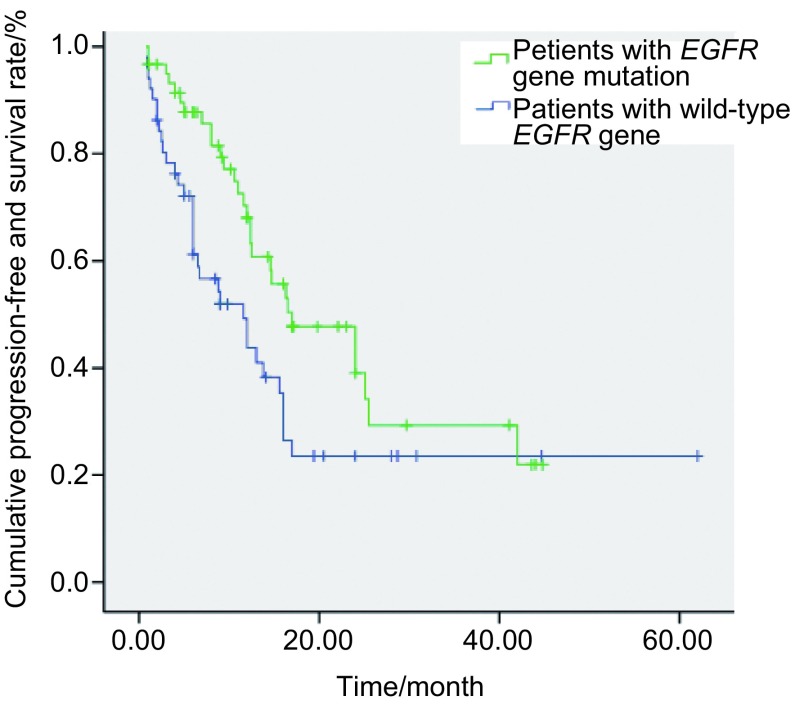
*EGFR*基因野生型和突变型患者的PFS曲线 The PFS curves of the *EGFR* mutation positive and negative patients. PFS: progression free survival.

### 2.4 OS和PFS的影响因素分析

分析性别、年龄、吸烟状况、ECOG评分、病理类型、既往化疗方案数、骨转移、脑转移、肝转移、其它肺叶转移、胸腔积液和*EGFR*基因突变对总生存期的影响。多因素分析结果见表 4。结果显示只有ECOG评分、病理类型和*EGFR*基因突变状态与OS相关。

**4:** 总生存期的*Cox*多因素分析 Multivariate analysis of over survial

Variables		HR	95%CI	Wald value	*P*
Gender	Male	1.722	0.722-4.102	1.503	0.220
Age	≤60	1.882	0.929-3.812	3.083	0.079
Smoking history	Never	0.718	0.277-1.863	0.465	0.496
ECOG PS	≥2	3.166	1.597-6.278	10.890	0.001
Pathology	Non-adenocarcinoma	4.681	1.957-11.201	12.027	0.001
Chemotherapy lines	≥2	1.624	0.844-3.123	2.110	0.146
Bone metastases	Yes	0.929	0.428-2.014	0.035	0.852
Brain metastases	Yes	0.716	0.276-1.861	0.469	0.493
Liver metastases	Yes	1.679	0.380-7.422	0.467	0.495
Other lobes metastases	Yes	1.232	0.624-2.433	0.363	0.548
Pleural effusion	Yes	0.757	0.360-1.593	0.537	0.464
*EGFR* mutation status	Wild type	1.912	1.022-3.575	4.113	0.043
Note: each group was compared with female, > 60, smokers, ECOG 0-1, adenocarcinoma, former chemotherapy lines ≤1, no bone matastases, no brain metastases, no liver metastases, no other lobes metastases, no pleural effusion, and *EGFR* mutation positive groups, respectively.					

分析性别、年龄、吸烟状况、ECOG评分、病理类型、既往化疗方案数、骨转移、脑转移、肝转移、其它肺叶转移、胸腔积液和*EGFR*基因突变对PFS的影响。多因素分析结果见表 5。结果显示ECOG评分、既往化疗方案数和*EGFR*基因突变与PFS明显相关。

**5:** PFS的*Cox*多因素分析 Multivariate analysis of progression free survival

Variables		HR	95%CI	Wald value	*P*
Gender	Male	1.799	0.910-3.559	2.849	0.091
Age	≤60	1.029	0.571-1.854	0.009	0.924
Smoking history	Never	0.779	0.363-1.672	0.410	0.522
ECOG PS	≥2	2.039	1.156-3.598	6.053	0.014
Pathology	Non-adenocarcinoma	1.517	0.713-3.225	1.170	0.279
Chemotherapy lines	≥2	1.808	1.010-3.236	3.968	0.046
Bone metastases	Yes	0.854	0.425-1.715	0.197	0.657
Brain metastases	Yes	0.564	0.240-1.328	1.717	0.190
Liver metastases	Yes	1.855	0.476-7.219	0.794	0.373
Other lobes metastases	Yes	1.039	0.580-1.863	0.017	0.897
Pleural effusion	Yes	1.239	0.694-2.210	0.524	0.469
*EGFR* mutation status	Negative	1.856	1.072-3.213	4.874	0.027
Note: each group was compared with female, > 60, smokers, ECOG 0-1, adenocarcinoma, former chemotherapy lines ≤1, no bone matastases, no brain metastases, no liver metastases, no other lobes metastases, no pleural effusion, and *EGFR* mutation positive groups, respectively.					

### 2.5 客观疗效的影响因素分析

分析客观疗效的影响因素包括年龄、性别、吸烟情况、ECOG评分、病理类型、既往化疗方案数、骨转移、脑转移、肝脏转移、其它肺叶转移、胸腔积液和*EGFR*基因突变状态（表 6），单因素分析显示吉非替尼的客观疗效与性别、吸烟情况、骨转移和*EGFR*基因突变状态明显相关。而多因素分析显示客观疗效只与突变状态（*χ*^2^=10.976, *P*=0.004）和吸烟情况（*χ*^2^=21.10, *P* < 0.001）相关，而其它因素无统计学意义。*EGFR*基因突变患者的客观有效率为43.3%（26/60），而疾病控制率为95%（57/60），高于*EGFR*基因野生型的患者，分别为21.6%（11/51）和76.5%（39/51）。

**6:** 患者临床特征对客观疗效的影响 The influence of clinical characteristics on objective effects

Characteristics		Objective response (*n*)			*χ*^2^	*P*
		PR	SD	PD		
Gender					17.37	< 0.001
	Male	8	32	12		
	Female	29	27	3		
Age					2.254	0.324
	≤60	20	27	10		
	> 60	17	32	5		
Smoking history					21.10	< 0.001
	Ever	2	20	10		
	Never	35	39	5		
ECOG PS					1.541	0.463
	0-1	26	36	8		
	≥2	11	23	7		
Pathology					5.237	0.073
	Adenocarcinoma	34	50	10		
	Non-adenocarcinoma	3	9	5		
Chemotherapy lines					1.900	0.387
	≤1	27	44	9		
	≥2	8	15	6		
Bone metastases					7.926	0.019
	Yes	17	12	3		
	No	20	47	12		
Brain metastases					0.331	0.848
	Yes	7	9	2		
	No	30	50	13		
Liver metastases					0.076	0.962
	Yes	2	4	1		
	No	35	35	14		
Other lobes metastases					0.195	0.907
	Yes	22	35	8		
	No	15	24	7		
Pleural effusion					2.433	0.296
	Yes	15	15	5		
	No	22	44	10		
*EGFR* mutation status					10.976	0.004
	Positive	26	31	3		
	Negative	11	28	12		
PR: partial response; SD: stable disease; PD: progressive disease.						

### 2.6 *EGFR*基因外显子19突变与外显子21突变的差别

外显子19缺失突变患者的OS为30.0个月（95%CI: 24.2-35.8）；外显子21点突变患者的OS为28.0个月（95%CI: 23.2-32.8），两者无明显差异（*χ*^2^=0.103, *P*=0.748）（图 3）。外显子19缺失突变患者的PFS为17.0个月（95%CI: 5.5-28.5）；外显子21点突变患者的PFS为16.5个月（95%CI: 3.9-29.1），两者无明显差异（*χ*^2^=0.060, *P*=0.807）（图 4）。

**3:**
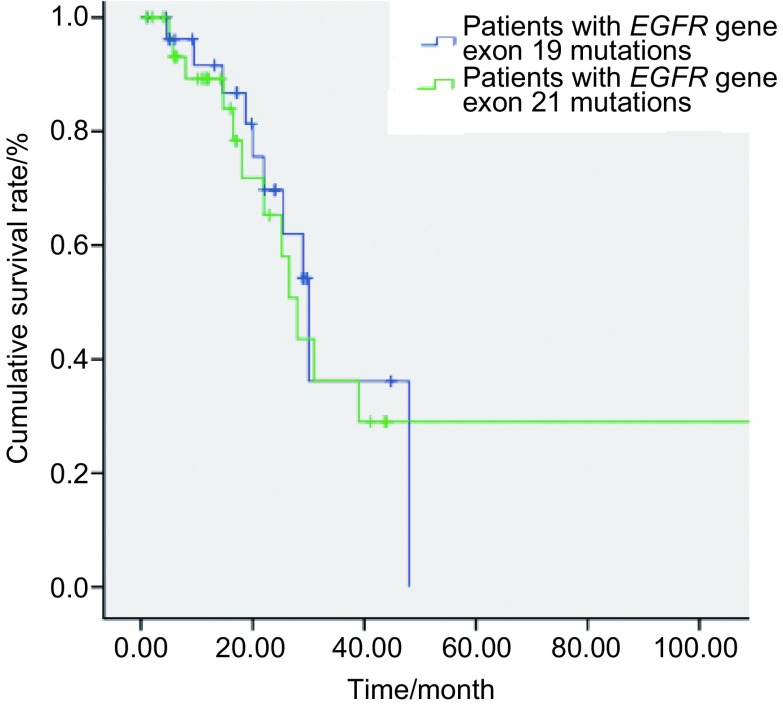
*EGFR*基因外显子19缺失突变和21点突变患者的OS曲线 The OS curves of the patients with *EGFR* exon 19 and 21 mutations

**4:**
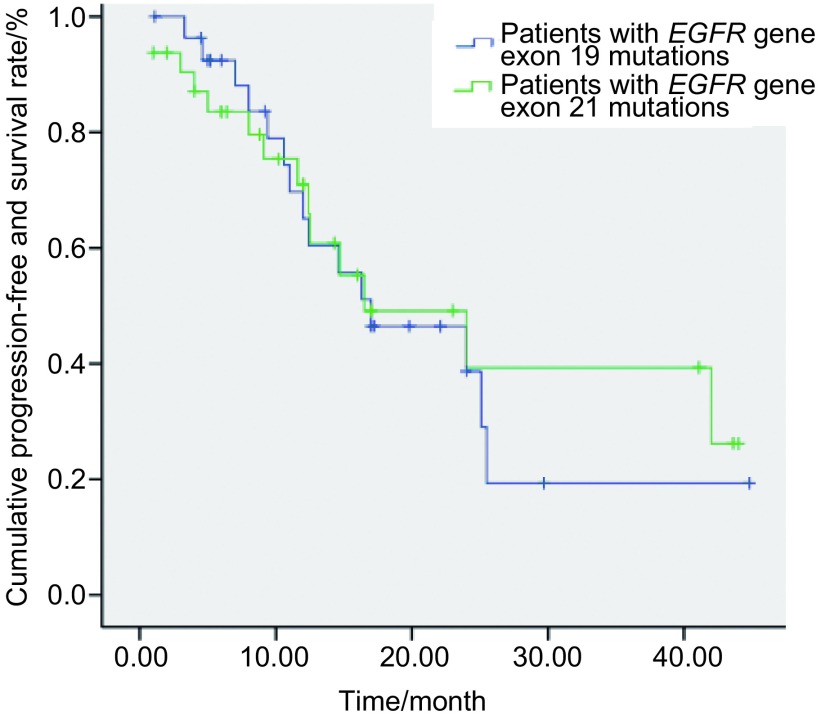
*EGFR*基因外显子19缺失突变和21点突变患者的PFS曲线 The PFS curves of the patients with *EGFR* exon 19 and 21 mutations

60例*EGFR*基因突变患者中外显子19缺失突变为28例，外显子21点突变为32例。外显子19缺失突变患者的客观有效率为42.8%（12/28），疾病稳定率为53.6%（15/28），疾病进展为3.6%（1/28）；而外显子21点突变患者的客观有效率为43.8%（14/32），疾病稳定率为50%（16/32），疾病进展为6.2%（2/32），两组的疗效无明显差异。

### 2.7 不良反应

患者使用吉非替尼的不良反应较少，主要为皮疹和腹泻，发生率分别为48.6%（54/111）和18.9%（21/111），但多数为轻度不良反应，不需要处理和停止吉非替尼治疗（表 7）。*EGFR*基因野生型和突变型患者的不良反应无明显差别。

**7:** 不良反应发生率 Rate of adverse events

Adverse events	Wild type	Mutation type	*χ*^2^	*P*
Rash	25 (49.0%)	29 (47.5%)	0. 077	0.781
Diarrhea	10 (19.6%)	11 (18.0%)	0.874	0.646
Loss of appetite	2 (4.0%)	1 (1.6%)	0.533	0.465
Elevated transaminase	2 (4.0%)	1 (1.6%)	0.533	0.465
Nausea/vomiting	0	2 (3.3%)	1.731	0.188

## 3 讨论

EGFR-TKI是近年来NSCLC治疗中最重要的进展，特别是发现*EGFR*基因突变与EGFR-TKI的疗效明显相关^[1, 2]^。普遍认为非鳞癌患者*EGFR*突变率较高，所有的非鳞癌患者都建议接受*EGFR*基因突变检测。本研究检测了160例非鳞癌的NSCLC，结果发现突变率达到55%，与相关文章报道相似^[4]^。IPASS试验^[5]^中进行*EGFR*基因突变检测的患者中突变阳性率为60%（261/437），该试验入组的患者都是腺癌和不吸烟或轻微吸烟的患者，而且*EGFR*基因突变中外显子19和外显子21突变所占的比例为90%以上。本研究发现在临床指标中，仅有病理类型和突变相关，腺癌的突变率高于大细胞肺癌和不能分型的NSCLC。

吉非替尼是最早研发的EGFR-TKI，本研究显示*EGFR*基因突变患者的PFS明显优于*EGFR*基因野生型患者，与文献报道一致。在IPASS试验^[5]^中发现*EGFR*基因突变阳性的患者的PFS为9.5个月，而野生型患者仅为1.5个月。这个差别远大于本研究，可能与IPASS试验是一线治疗试验，即患者之前未接受过化疗，而本研究的患者大部分都是化疗后才接受了吉非替尼的治疗有关。多数文章^[5-12]^均报道*EGFR*基因突变不仅是EGFR-TKI的疗效预测因素，也是患者生存的预后因素。在吉非替尼的一线治疗IPASS试验^[5]^中，*EGFR*基因突变患者的总生存期为21.6个月，而野生型为11.2个月；二线治疗INTEREST试验^[6]^中分别为14.2个月和6.4个月。本研究中两组的总生存期分别为29.0个月和21.0个月，虽然两组总生存期有8个月的差异，但未达到统计学差异。其原因可能为两组的总生存期都很长并且两组患者例数偏少。

多因素分析显示OS的影响因素为ECOG评分和病理类型，PFS的影响因素为ECOG评分、既往化疗方案数和*EGFR*基因突变。提示了虽然吉非替尼可以用于ECOG评分3分-4分的患者和三线甚至更晚使用仍然有效，但是除了*EGFR*基因突变外，ECOG评分越差和既往的化疗方案越多，则吉非替尼治疗的PFS越差，患者的获益越小。特别是ECOG评分与患者生存也相关，提示要在患者一般情况好时就使用吉非替尼，而不是等到患者情况恶化时。

本研究中*EGFR*基因突变患者的客观有效率为43.3%，高于*EGFR*基因野生型的患者（21.6%）。多篇文献报道对于最初*EGFR*基因突变阳性的人群，EGFR-TKIs作为二/三线治疗和一线治疗的客观有效率是有差异的，分别为30%-40%^[6-10]^和70%^[5, 11, 12]^，这与本研究中*EGFR*基因突变患者的客观有效率为43.3%是一致的。化疗后*EGFR*基因突变患者EGFR-TKI疗效下降的原因不清楚。韩如冰等^[3]^报道化疗前后外周血*EGFR*基因突变状态一致率仅为54.5%（18/33），部分患者化疗前*EGFR*基因突变阴性变为阳性，部分患者化疗前阳性变为阴性。Chin等^[13]^将含有*EGFR*外显子19缺失突变的NSCLC细胞系PC9细胞系经过铂类化疗药物处理耐药后，再使用厄洛替尼，发现厄洛替尼的敏感性降低，说明化疗可能在酪氨酸激酶激活通路上亦有作用。因此二/三线EGFR-TKIs治疗有效率的降低可能部分归因于由于化疗导致的EGFR-TKIs耐药，具体机制有待进一步临床研究证实。

近90%的*EGFR*基因突变集中于外显子19的缺失突变和外显子21点突变，本研究显示两种突变的PFS和OS均无差别。在IPASS试验^[5]^中，接受吉非替尼的*EGFR*基因外显子19缺失突变患者和外显子21点突变患者分别为66例和64例，两种突变患者的PFS均大约为9个月。NEJ002试验^[11]^中，接受吉非替尼的*EGFR*基因外显子19缺失突变患者和21外显子点突变患者分别为58例和49例，两种突变患者的PFS分别为11.5个月和10.8个月。上述两试验与本研究相似，提示突变类型与治疗效果无明显关系。*EGFR*基因外显子19缺失突变和外显子21点突变的客观有效率也无明显差别。

由于本研究是一项回顾性研究，结果也可能存在着一些偏倚。综上所述，晚期非鳞癌NSCLC *EGFR*基因突变患者的PFS明显优于野生型患者，OS有延长趋势。*EGFR*基因不同突变类型的PFS和OS均无差别。

## References

[1] Lynch TJ, Bell DW, Sordella R (2004). Activating mutations in the epidermal growth factor receptor underlying responsiveness of non-small cell lung cancer to gefitinib. N Engl J Med.

[2] Paez JG, Janne PA, Lee JC (2004). *EGFR* mutations in lung cancer: correlation with clinical response to gefitinib therapy. Science.

[3] Han RB, Zhong W, Zhao J (2011). Comparison of serum *EGFR* gene mutation status in advanced lung cancer patients before and after chemotherapy. Zhongguo Fei Ai Za Zhi.

[4] Rafael R, Moran T, Queralt C (2009). Screening for epiderminal growth factor receptor mutations in lung cancer. N Engl J Med.

[5] Mok TS, Wu YL, Thongprasert S (2009). Gefitinib or carboplatin-paclitaxel in pulmonary adenocarcinoma. N Engl J Med.

[6] Douillard JY, Shepherd FA (2010). Molecular predictors of outcome with gefitinib and docetaxel in previously treated non-small cell lung cancer: data from the randomized phase Ⅲ INTEREST trial. J Clin Oncol.

[7] Maruyama R, Nishiwaki Y, Tamura T (2008). Phase Ⅲ study, Ⅴ-15-32, of gefitinib versus docetaxel in previously treated Japanese patients with non-small cell lung cancer. J Clin Oncol.

[8] Thatcher N, Chang A, Parikh P (2005). Gefitinib plus best supportive care in previously treated patients with refractory advanced non-small cell lung cancer: results from a randomised, placebo-controlled, multicentre study (Iressa Survival Evaluation in Lung Cancer). Lancet.

[9] Hirsch FR, Varella-Garcia M, Bunn Jr PA (2006). Molecular predictors of outcome with gefitinib in a phase Ⅲ placebo-controlled study in advanced non-small cell lung cancer. J Clin Oncol.

[10] Zhu CQ, da Cunha Santos G, Ding K (2008). Role of KRAS and EGFR as biomarkers of response to erlotinib in National Cancer Institute of Canada Clinical Trials Group Study BR.21. J Clin Oncol.

[11] Maemondo M, Inoue A, Kobayashi K (2010). Gefitinib or chemotherapy for non-small cell lung cancer with mutated *EGFR*. N Engl J Med.

[12] Mitsudomi T, Morita S, Yatabe Y (2010). Gefitinib versus cisplatin plus docetaxel in patients with non-small cell lung cancer harbouring mutations of the epidermal growth factor receptor (WJTOG3405): an open label, randomised phase 3 trial. Lancet Oncol.

[13] Chin TM, Quinlan MP, Singh A (2008). Reduced erlotinib sensitivity of epidermal growth factor receptor-mutant non-small cell lung cancer following cisplatin exposure: a cell culture model of second-line erlotinib treatment. Clin Cancer Res.

